# MIT-001, a Mitochondria-Targeted ROS Scavenger, Ameliorates DSS-Induced Colitis and Is Associated with Reduced HMGB1 and IL-1β Expression

**DOI:** 10.3390/ijms27136051

**Published:** 2026-07-06

**Authors:** Dongwoo Kim, Soon Ha Kim, Jung Wan Choe, Seung Young Kim, Jong Jin Hyun, Sung Woo Jung, Young Kul Jung, Hyung Joon Yim, Ja Seol Koo

**Affiliations:** 1 Department of Internal Medicine, Korea University College of Medicine, Seoul 02841, Republic of Korea; blustick@korea.ac.kr (D.K.);; 2 Division of Gastroenterology and Hepatology, Department of Internal Medicine, Korea University Ansan Hospital, Ansan 15355, Republic of Korea; 3 Mitoimmune Therapeutics Inc., Seoul 05836, Republic of Korea

**Keywords:** inflammatory bowel disease, ulcerative colitis, necrosis, HMGB1, mitochondrial reactive oxygen species, MIT-001

## Abstract

Inflammatory bowel disease (IBD) is characterized by chronic intestinal inflammation in which excessive cell death and the release of damage-associated molecular patterns (DAMPs) such as high-mobility group box 1 (HMGB1) amplify mucosal injury. Although necrosis—particularly regulated forms including necroptosis and ferroptosis—has emerged as a contributor to IBD pathogenesis, the therapeutic potential of targeting necrotic cell death remains incompletely explored. We investigated whether MIT-001 (previously known as NecroX-7), a mitochondria-targeted reactive oxygen species (ROS) scavenger with anti-necrotic activity, ameliorates intestinal inflammation in an acute dextran sulfate sodium (DSS)-induced colitis model. In vitro, MIT-001 reduced hydrogen peroxide-induced necrotic cell death in IEC-18 intestinal epithelial cells and was associated with a qualitative reduction in the 55-kDa cleaved poly(ADP-ribose) polymerase-1 (PARP-1) fragment (a marker of necrosis), with no apparent change in the apoptosis-related 89-kDa fragment. In vivo, oral administration of MIT-001 to C57BL/6 mice with DSS-induced colitis was associated with preservation of colon length, reduced histological injury, and a marked decrease in HMGB1-positive cells in colonic tissue. Among pro-inflammatory cytokines, IL-1β expression was significantly reduced, while IL-12, monocyte chemoattractant protein-1 (MCP-1), and TNF-α showed non-significant downward trends. These findings indicate that MIT-001 ameliorates DSS-induced colitis in association with reduced HMGB1 and IL-1β expression, supporting further investigation of mitochondria-targeted anti-necrotic strategies as a potential adjunctive approach in IBD.

## 1. Introduction

Inflammatory bowel disease (IBD), encompassing ulcerative colitis and Crohn’s disease, is characterized by chronic, uncontrolled inflammation, leading to progressive intestinal damage [1]. Current therapeutic strategies primarily focus on suppressing excessive inflammation using agents such as 5-aminosalicylic acid, corticosteroids, and immunomodulators including azathioprine. With advances in the understanding of inflammatory pathways, biologic agents targeting specific mediators—such as tumor necrosis factor-α (TNF-α), integrins, and interleukin (IL)-12/23—as well as small-molecule inhibitors, including Janus kinase (JAK) inhibitors, have been developed and are widely used in clinical practice [2,3]. However, therapeutic response rates remain limited to approximately 50% in patients with moderate-to-severe IBD, highlighting the need for alternative therapeutic approaches [4,5].

Excessive inflammatory responses lead to the release of reactive mediators, including cytokines and reactive oxygen species (ROS), from activated inflammatory cells, resulting in damage to surrounding tissues and subsequent cell death [6]. Cell death occurs through two major pathways: apoptosis and necrosis [7]. Apoptosis is a regulated process that proceeds without eliciting significant inflammatory responses, whereas necrosis is characterized by disruption of the plasma membrane and release of intracellular contents, triggering a robust inflammatory response and a self-amplifying cycle of inflammation and necrosis. Given that prominent inflammation and mucosal ulceration are key endoscopic features of IBD, necrosis has been suggested as a major form of cell death contributing to disease pathogenesis. A regulated form of necrosis, termed necroptosis, has been described and shares morphological features with necrosis [8]. Unlike unregulated necrosis, necroptosis is a tightly controlled process that can be therapeutically targeted. Animal studies have demonstrated that necroptosis of intestinal epithelial cells can induce intestinal inflammation resembling that observed in IBD [9,10], and increased expression of key necroptosis mediators, including receptor-interacting protein kinase 3 (RIPK3) and mixed-lineage kinase domain-like protein (MLKL), has been reported in patients with IBD [11]; the contribution of necroptosis and other regulated cell death programs to intestinal inflammation has been increasingly emphasized in recent work [12,13].

Beyond necroptosis, ferroptosis—an iron-dependent form of regulated cell death—has also been implicated in intestinal epithelial damage in IBD [14,15,16]. Notably, both necrosis and ferroptosis converge mechanistically on mitochondrial ROS generation and the release of DAMPs such as HMGB1, suggesting that mitochondria-targeted ROS scavengers may modulate multiple regulated necrotic cell death pathways relevant to colitis. However, therapeutic strategies specifically targeting mitochondrial ROS-mediated necrotic cell death remain largely unexplored in IBD.

In the present study, we investigated whether MIT-001 (previously known as NecroX-7), a mitochondria-targeted ROS scavenger with previously reported anti-necrotic activity [17,18,19], ameliorates intestinal inflammation in vitro and in an acute DSS-induced colitis model. MIT-001 is a cyclopentylamino carboxymethylthiazolylindole compound developed as a mitochondria-targeted ROS and peroxynitrite scavenger; it has been reported to attenuate necrotic cell death by inhibiting mitochondrial calcium uniporter activity, scavenging mitochondrial free radicals, and stabilizing mitochondrial membrane potential, with limited effect on apoptosis [17,18,19]. More recently, it has been reported to inhibit ferroptosis [20].

## 2. Results

### 2.1. MIT-001 Reduces Necrotic Cell Death in IEC-18 Cells

**Figure 1:**
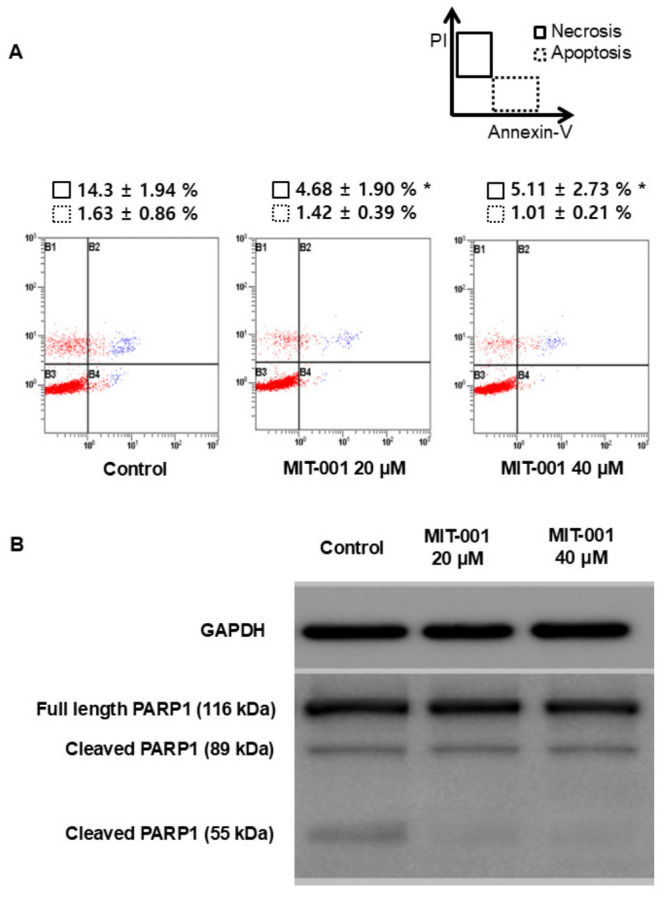
MIT-001 reduces necrotic cell death in IEC-18 cells. (**A**) Necrotic (PI-positive, Annexin V-negative) and apoptotic (Annexin V-positive) cell fractions assessed by flow cytometry. Dot colors indicate relative cell density (red, higher density; blue, lower density). (**B**) PARP-1 cleavage analyzed by Western blot. * *p* < 0.05 versus control. Data in (**A**) are presented as mean ± SEM; two-group comparisons were performed using the Mann–Whitney U test. The Western blot in (**B**) is representative and was assessed qualitatively. The original, uncropped blot images are provided in Figure S1.

We first evaluated whether MIT-001 reduces necrotic cell death in IEC-18 intestinal epithelial cells. Exposure to 500 μM hydrogen peroxide for 4 h induced necrotic cell death in approximately 14.3% of vehicle-treated cells (Figure 1A). Pretreatment with MIT-001 significantly reduced the proportion of necrotic cells (Annexin V-negative, propidium iodide [PI]-positive), falling by approximately two-thirds compared with the vehicle control (20 μM MIT-001 vs. control: 4.68 ± 1.90% vs. 14.3 ± 1.94%; *p* = 0.029, Mann–Whitney U test). No additional inhibitory effect was observed at higher concentrations (20 μM vs. 40 μM: 4.68 ± 1.90% vs. 5.11 ± 2.73%; *p* = 0.564).

To further characterize the type of cell death targeted by MIT-001, we examined PARP-1 cleavage patterns by Western blot analysis (Figure 1B). In a representative blot, the 116-kDa full-length PARP-1 and the 89-kDa apoptosis-associated fragment showed comparable band intensities across conditions, whereas the 55-kDa cleaved PARP-1 fragment, a marker of necrosis, appeared reduced in MIT-001-treated cells relative to vehicle controls. As this Western blot analysis was qualitative, these observations should be interpreted as supportive rather than confirmatory; nonetheless, together with the flow cytometric findings, they are consistent with preferential attenuation of necrotic, rather than apoptotic, cell death in intestinal epithelial cells.

### 2.2. MIT-001 Ameliorates Colonic Inflammation in an Acute DSS-Induced Colitis Model

**Figure 2:**
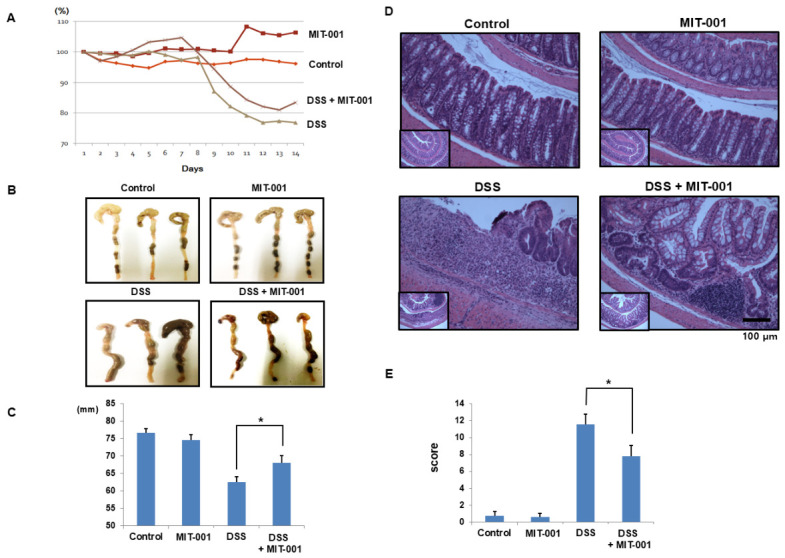
MIT-001 ameliorates acute DSS-induced colitis. (**A**) Body weight changes over 14 days. (**B**) Representative colons and (**C**) colon length. (**D**) Representative colonic histology and (**E**) histological injury scores. * *p* < 0.05. Data are presented as mean ± SEM. For colon length (**C**), *n* = 3 (control), 3 (MIT-001), 7 (DSS), and 5 (DSS + MIT-001); for histological injury scores (**E**), *n* = 4 (control), 4 (MIT-001), 8 (DSS), and 6 (DSS + MIT-001). Two-group comparisons were performed using the Mann–Whitney U test. In (**D**), scale bar = 100 μm. In (**A**), body weight is expressed as the percentage change from baseline; endpoint (2-week) body weight loss was compared between groups using the Mann–Whitney U test.

Body weight changes across treatment groups are shown in Figure 2A. In the DSS group, body weight loss began on day 7 and reached approximately 20% of baseline by 2 weeks. Although the DSS + MIT-001 group showed approximately 4% less body weight loss than the DSS group, this difference did not reach statistical significance (DSS + MIT-001 vs. DSS: −18.7 ± 3.2% vs. −23.1 ± 2.8%; *p* = 0.640).

For the in vivo experiments, mice were allocated to four groups (control and MIT-001 groups, *n* = 4 each; DSS and DSS + MIT-001 groups, *n* = 8 each). Two mice in the DSS + MIT-001 group died during the study, and the remaining animals were analyzed. Colon length, a surrogate marker of colonic inflammation, was significantly preserved in the MIT-001-treated group compared with the DSS group (Figure 2B,C; DSS + MIT-001 vs. DSS: 68.0 ± 2.10 mm vs. 62.6 ± 1.45 mm; *p* = 0.048). Histological examination revealed that DSS-treated mice exhibited marked inflammatory cell infiltration, epithelial desquamation, and goblet cell depletion, all of which were attenuated by MIT-001 treatment (Figure 2D). Semiquantitative histological scoring confirmed a significant reduction in colonic injury in the DSS + MIT-001 group (Figure 2E; DSS + MIT-001 vs. DSS: 7.78 ± 1.31 vs. 11.6 ± 1.17; *p* = 0.038).

### 2.3. MIT-001 Decreases HMGB1 Expression in Colonic Tissue

**Figure 3:**
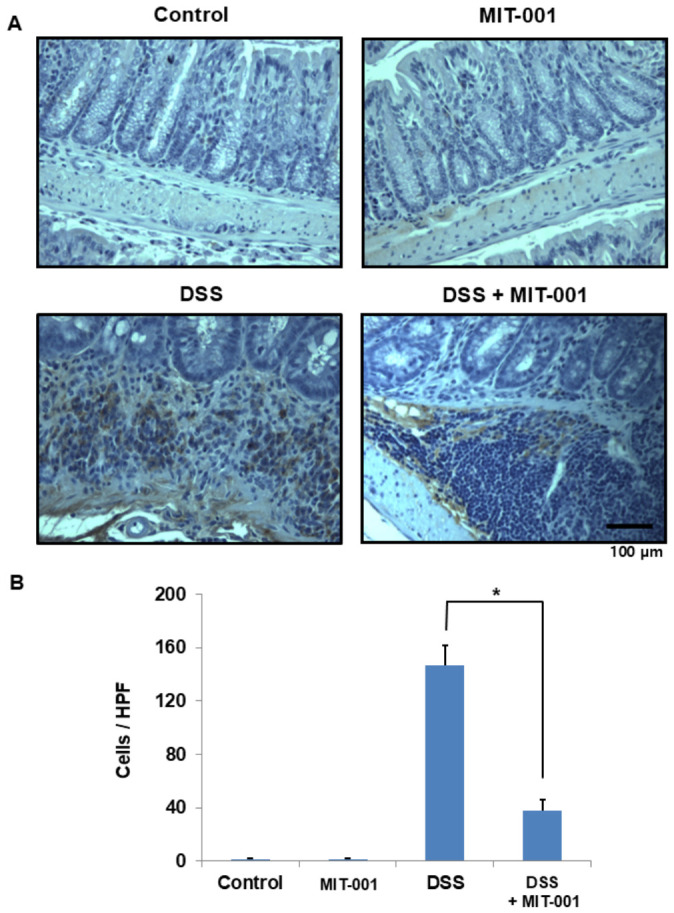
MIT-001 decreases HMGB1 expression in DSS-induced colitis. (**A**) Representative HMGB1 immunohistochemical staining of colonic tissue. Brown staining indicates HMGB1-positive cells; blue (hematoxylin) counterstaining indicates nuclei. (**B**) Quantification of HMGB1-positive cells per high-power field (HPF). * *p* < 0.05. HMGB1-positive cells were counted in representative high-power fields per animal and are expressed as cells per HPF (mean ± SEM); *n* = 8 (DSS) and *n* = 6 (DSS + MIT-001); control and MIT-001 groups, *n* = 4 each. Two-group comparisons were performed using the Mann–Whitney U test. In (**A**), scale bar = 100 μm and applies to all panels. The original immunohistochemistry images are provided in Figure S1.

To assess HMGB1 involvement in DSS-induced colitis and the effect of MIT-001 on HMGB1 expression, we performed immunohistochemical staining of colonic sections. HMGB1-positive cells were markedly increased in DSS-treated mice and were predominantly localized to areas of inflammation (Figure 3A). MIT-001 treatment significantly reduced the number of HMGB1-positive cells (Figure 3A,B; DSS + MIT-001 vs. DSS: 37.5 ± 8.63 vs. 146.5 ± 15.1 cells per high-power field; *p* = 0.021).

### 2.4. MIT-001 Reduces Pro-Inflammatory Cytokine Expression

**Figure 4:**
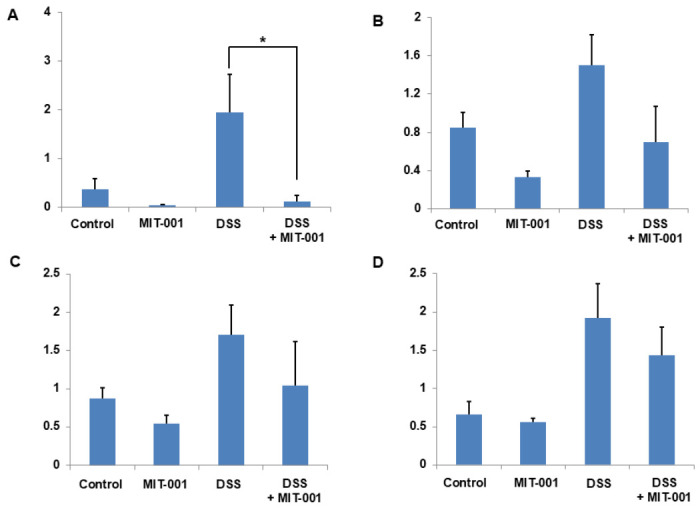
Effect of MIT-001 on colonic inflammatory cytokine expression. Colonic mRNA levels of (**A**) IL-1β, (**B**) IL-12, (**C**) MCP-1, and (**D**) TNF-α. * *p* < 0.05. Data are presented as mean ± SEM; owing to occasional technical sample losses, the number of animals analyzed per cytokine ranged from 2 to 4 (control), 3 to 4 (MIT-001), 7 to 8 (DSS), and 4 to 6 (DSS + MIT-001), depending on the cytokine. Two-group comparisons were performed using the Mann–Whitney U test, without correction for multiple comparisons. mRNA levels were quantified by real-time PCR in proximal and distal colon tissue using the comparative Ct (ΔΔCt) method, with glyceraldehyde-3-phosphate dehydrogenase (GAPDH) as the endogenous reference gene.

Expression levels of pro-inflammatory cytokines in colonic tissue are shown in Figure 4. IL-1β, IL-12, MCP-1, and TNF-α mRNA levels were significantly elevated in the DSS group compared with controls (*p* < 0.05). MIT-001 treatment was associated with a significant reduction in IL-1β expression (DSS + MIT-001 vs. DSS: 0.12 ± 0.12 vs. 1.95 ± 0.77; *p* = 0.011). IL-12, MCP-1, and TNF-α showed a downward trend in the DSS + MIT-001 group, but the differences were not statistically significant (IL-12: 0.70 ± 0.37 vs. 1.50 ± 0.32, *p* = 0.057; MCP-1: 1.04 ± 0.58 vs. 1.71 ± 0.38, *p* = 0.107; TNF-α: 1.43 ± 0.37 vs. 1.92 ± 0.45, *p* = 0.668).

## 3. Discussion

In this study, MIT-001, a mitochondria-targeted ROS scavenger with anti-necrotic activity, reduced hydrogen peroxide-induced necrotic cell death in IEC-18 cells (Figure 1) and ameliorated colonic inflammation in an acute DSS-induced colitis model. MIT-001 treatment was associated with a marked decrease in HMGB1-positive cells (Figure 3) and a significant reduction in IL-1β expression (Figure 4), supporting the involvement of mitochondrial ROS-mediated necrotic cell death and HMGB1-related inflammatory signaling in DSS-induced colitis. Importantly, the present data establish an association rather than a direct causal relationship, as dedicated mechanistic experiments (e.g., HMGB1 blockade or rescue, RIPK3/MLKL pathway analysis) were not performed.

The DSS-induced colitis model is widely used in IBD research. DSS exerts a direct toxic effect on the colonic epithelium and increases intestinal permeability, thereby facilitating activation of submucosal macrophages via Toll-like receptors and the nuclear factor-κB (NF-κB) pathway [21], leading to the production of pro-inflammatory cytokines, including IL-1β, and initiating an intestinal inflammatory cascade [22,23]. Activated inflammatory cells generate ROS and oxidative stress, which result in membrane damage and dysregulation of intracellular Ca^2+^, ultimately inducing necrotic cell death [24,25,26]. MIT-001 is a multifunctional small molecule that scavenges mitochondrial ROS and peroxynitrite, inhibits mitochondrial calcium uniporter activity, and stabilizes mitochondrial membrane potential, thereby attenuating necrotic and necroptotic cell death across diverse injury models [17,18,19]. More recently, MIT-001 has also been identified as an inhibitor of ferroptosis, broadening its anti-cell-death spectrum to include lipid peroxidation-dependent regulated cell death [20]. In the present study, MIT-001 attenuated DSS-induced colonic inflammation, consistent with a contribution of mitochondrial ROS-mediated cell death to the pathophysiology of intestinal inflammation. Because MIT-001 has multiple pharmacological effects, the observed anti-inflammatory action likely reflects the combined consequences of mitochondrial ROS scavenging, attenuation of necrotic and necroptotic cell death, and inhibition of downstream DAMP release rather than a single, isolated mechanism.

HMGB1 is a nuclear non-histone protein involved in gene transcription and DNA repair. It can be passively released into the extracellular space during necrotic cell death or actively secreted by activated inflammatory cells [27]. Extracellular HMGB1 acts as a DAMP and directly induces the production of pro-inflammatory cytokines, including IL-1β, from peripheral blood mononuclear cells [28]. HMGB1 also promotes inflammation indirectly by recruiting inflammatory cells to sites of injury [29]; these cells can in turn be activated and release additional HMGB1 and cytokines, amplifying inflammation in a self-sustaining cycle [30]. Previous studies have shown that HMGB1 contributes to the pathogenesis of inflammatory diseases, including sepsis, hepatitis, and systemic lupus erythematosus [31,32,33]. In pediatric patients with IBD, fecal HMGB1 levels are elevated and correlate with colonic inflammation [34]. Experimental studies have also shown that HMGB1 expression increases in DSS-induced colitis and that anti-HMGB1 antibody treatment attenuates colonic inflammation [35].

In the present study, DSS-treated mice showed a marked increase in HMGB1-positive cells predominantly localized to severely inflamed mucosal and submucosal areas, whereas MIT-001 treatment was associated with both reduced HMGB1 expression (Figure 3) and attenuated colonic inflammation (Figure 2). These findings are consistent with a model in which mitochondrial ROS-mediated necrotic cell death contributes to HMGB1 accumulation in the inflamed colon and in which suppression of this cell-death axis may disrupt the self-amplifying cycle between inflammation and necrosis. Among the pro-inflammatory cytokines examined, only IL-1β was significantly reduced (Figure 4). This selective reduction in IL-1β mRNA is consistent with prior reports linking necrosis-driven HMGB1 signaling to IL-1β expression [22]; however, because only IL-1β mRNA was measured, our data do not allow conclusions regarding inflammasome activation or IL-1β protein maturation, whereas IL-12, MCP-1, and TNF-α displayed only non-significant downward trends. The absence of a significant TNF-α reduction—despite TNF-α being a major therapeutic target in IBD—suggests that mitochondria-targeted anti-necrotic therapy is unlikely to replace established anti-TNF biologics but may instead complement existing strategies by suppressing an upstream cell-death-driven inflammatory amplifier [36]. Taken together, the present data indicate that MIT-001 significantly preserved colon length and reduced the histological injury score, indicating attenuation of tissue-level mucosal damage, whereas body weight loss was not significantly improved and, among the cytokines examined, only IL-1β was significantly reduced. This pattern suggests that the protective effect of MIT-001 in acute DSS colitis, although consistent across several independent readouts, is modest and selective, being more apparent in tissue-level injury and in the HMGB1/IL-1β axis than in systemic measures such as body weight or the broader cytokine panel.

Our findings are broadly consistent with previous reports on other mitochondria-targeted antioxidants in experimental colitis. MitoQ, a mitochondria-targeted ubiquinone derivative, ameliorated DSS-induced colitis with reduced colon-length shortening, lower histological scores, and suppression of IL-1β, and has since progressed to clinical evaluation in ulcerative colitis [37]. Other mitochondria-targeted agents, including SkQ1 and MitoTEMPO, have likewise shown protective effects on colonic inflammation and epithelial barrier function in DSS models [38,39]. The selective reduction in IL-1β observed with MIT-001 parallels the cytokine-suppressive effect reported for MitoQ and supports the broader concept that limiting mitochondrial ROS can attenuate colonic inflammation. At the same time, the comparatively modest in vivo effect of MIT-001 in our acute model, together with reports of variable MitoQ efficacy across different colitis protocols, underscores that the magnitude of benefit from mitochondria-targeted antioxidants is model- and protocol-dependent.

This study has several important limitations. First, the conclusions reflect an association between MIT-001 treatment and reduced HMGB1 and IL-1β expression rather than a definitive causal pathway; confirmatory experiments such as HMGB1 blockade or knockdown, recombinant HMGB1 rescue, and downstream receptor for advanced glycation end products (RAGE)/Toll-like receptor 4 (TLR4)/ NF-κB pathway analyses will be required. Second, although the PARP-1 cleavage pattern and Annexin V/PI staining support necrotic cell death as the primary readout, specific markers of necroptosis (e.g., RIPK1/RIPK3/MLKL phosphorylation) and ferroptosis (e.g., glutathione peroxidase 4 (GPX4), lipid peroxidation) were not assessed, so the relative contributions of these regulated cell-death pathways remain to be delineated. Third, immunohistochemistry quantifies total HMGB1 and cannot distinguish nuclear, cytoplasmic, or extracellularly released HMGB1; Western blot, ELISA, and co-localization studies will be needed to clarify its cellular source and functional state. Fourth, the sample size was modest (*n* = 4–8 per group after attrition), several findings were of borderline significance, and multiple cytokine comparisons warrant cautious interpretation. Fifth, only acute DSS colitis in male mice was studied, and MIT-001 was given before and during disease induction; chronic or genetically driven models, inclusion of female animals, and therapeutic-dosing designs that begin after colitis is established will be needed to assess generalizability and clinical relevance. Finally, only a single oral dose was tested without a comparator necrosis inhibitor, and pharmacokinetics, intestinal microbiota, systemic toxicity, and epithelial barrier integrity were not evaluated; these should be addressed in future translational studies.

## 4. Materials and Methods

### 4.1. MIT-001

MIT-001 (MitoImmune Therapeutics Inc., Seoul, Republic of Korea), previously known as NecroX-7, was provided by the manufacturer. For in vitro experiments, MIT-001 was dissolved in normal saline and applied to cells at final concentrations of 20 μM or 40 μM, with normal saline serving as the vehicle control. For in vivo experiments, MIT-001 was dissolved in normal saline and administered at 30 mg/kg by once-daily oral gavage, with an equal volume of normal saline used as the vehicle control.

### 4.2. Cell Culture and In Vitro Assessment of Necrosis Inhibition

Rat intestinal epithelial cells (IEC-18; American Type Culture Collection [ATCC], Manassas, VA, USA) were cultured in Dulbecco’s modified Eagle’s medium (DMEM)/F12 (1:1; Gibco, Thermo Fisher Scientific, Waltham, MA, USA) supplemented with 10% fetal bovine serum (Sigma-Aldrich, St. Louis, MO, USA) at 37 °C in a humidified 5% CO_2_ atmosphere. To induce necrosis, cells were exposed to 500 μM hydrogen peroxide (Sigma-Aldrich, St. Louis, MO, USA) for 4 h. MIT-001 (20 μM or 40 μM) or vehicle was added 1 h before hydrogen peroxide exposure. Cell death was assessed by Annexin V–propidium iodide (PI) staining (BD Pharmingen, San Diego, CA, USA), followed by flow cytometric analysis on a FACSCalibur flow cytometer (BD Biosciences, San Jose, CA, USA). Cells negative for Annexin V and positive for PI were defined as necrotic.

To confirm the inhibitory effect of MIT-001 on necrotic cell death, PARP-1 cleavage patterns were analyzed by Western blot. PARP-1 is cleaved at distinct sites depending on the mode of cell death, generating fragments of different molecular weights [40]; during apoptosis, PARP-1 is cleaved into 89-kDa and 24-kDa fragments, whereas necrotic cell death is characterized by a 55-kDa fragment. Equal amounts of protein (20 μg, quantified by Bradford assay) were separated by 8% SDS-PAGE and transferred onto a polyvinylidene fluoride membrane (Millipore, Schwalbach, Germany). Membranes were incubated with primary antibodies against full-length and cleaved PARP-1 (Cell Signaling Technology, Danvers, MA, USA), followed by peroxidase-conjugated secondary antibodies, and visualized using an enhanced chemiluminescence detection kit (Promega, Madison, WI, USA). PARP-1 cleavage was evaluated qualitatively from representative immunoblots; densitometric quantification was not performed. The molecular weights of the cleaved fragments were assigned relative to the full-length PARP-1 band (116 kDa).

### 4.3. Acute DSS-Induced Colitis Model

**Figure 5:**
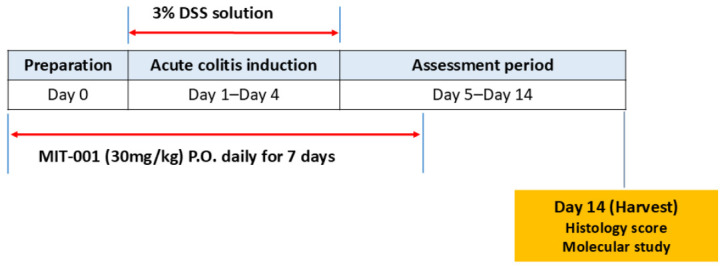
Schematic of the experimental design for the acute DSS-induced colitis model.

Male C57BL/6 mice (7–8 weeks old; Raonbio, Yongin, Republic of Korea; non-specific-pathogen-free) were used for in vivo experiments. All animal procedures were approved by the Institutional Animal Care and Use Committee of Korea University College of Medicine (Approval No. KOREA-2016-0183; approved 28 September 2016) and were conducted in accordance with relevant institutional guidelines and the ARRIVE guidelines. Animals were acclimatized for 1 week before the experiments and were housed 2 per cage under controlled conditions (temperature 20–26 °C, relative humidity 40–60%, 12 h light/12 h dark cycle) with food and water provided ad libitum; no environmental enrichment was provided. Mice were randomly assigned, using a random number table, to four groups: a vehicle-treated control group (control, *n* = 4), a MIT-001-only group (MIT-001, *n* = 4), a DSS-only group (DSS, *n* = 8), and a DSS + MIT-001 group (*n* = 8), for a total of 24 animals. Group sizes were based on prior experience with the acute DSS colitis model; no a priori power calculation was performed. Potential confounders such as cage location and the order of treatment and measurement were not specifically controlled. During the study, 2 mice in the DSS + MIT-001 group died; no deaths occurred in the other groups. General condition and body weight were monitored daily, and no humane endpoints were predefined. The exact number of animals analyzed for each outcome is reported in the corresponding figure legends. Acute colitis was induced by ad libitum administration of 3% (*w*/*v*) dextran sulfate sodium (DSS, molecular weight 36,000–50,000 Da) in drinking water for 4 days. Vehicle or MIT-001 (30 mg/kg) was administered once daily by oral gavage starting 1 day before DSS administration and continuing through day 7 (Figure 5). Body weight was monitored daily. Mice were euthanized by CO_2_ inhalation followed by cervical dislocation on day 14, and colon length (from anus to cecum) was measured. Proximal and distal colon tissues were immediately snap-frozen in liquid nitrogen for real-time PCR analysis. For histological evaluation, colons were harvested, rolled from the distal to proximal end (“Swiss roll”), fixed in 4% paraformaldehyde, and embedded in paraffin.

### 4.4. Histologic and Immunohistochemical Evaluation

Paraffin-embedded colon tissues were sectioned at 4 μm, deparaffinized in xylene, and stained with hematoxylin and eosin. Sections were examined by light microscopy at ×40, ×100, and ×200 magnification. Two physicians blinded to group assignment independently scored intestinal injury based on five parameters—inflammation severity, extent, regeneration, crypt damage, and percentage involvement—as detailed in Table 1. Scores from three colonic segments (proximal, mid, and distal) were averaged for each animal. For HMGB1 immunohistochemistry, sections were incubated with an anti-HMGB1 primary antibody, and HMGB1-positive cells (defined as cells displaying either cytoplasmic or nuclear staining) were counted per high-power field. HMGB1 immunohistochemistry quantifies total HMGB1 staining and does not differentiate among nuclear, cytoplasmic, and extracellularly released pools.

### 4.5. Quantification of Inflammatory Cytokines by Real-Time PCR

Proximal and distal colon segments were homogenized using pellet pestles (Sigma-Aldrich, St. Louis, MO, USA), and total RNA was extracted with TRIzol reagent (Sigma-Aldrich). RNA concentration was measured using Take3 and Take3 Trio microvolume plates (BioTek, Winooski, VT, USA). Complementary DNA was synthesized from 100 ng of total RNA using the GoScript Reverse Transcription System (Promega, Madison, WI, USA). Real-time PCR was performed with the LightCycler FastStart DNA Master SYBR Green I kit (Roche, Basel, Switzerland) on a LightCycler 480 instrument (Roche, Basel, Switzerland) under the following thermal program: 10 min at 95 °C for polymerase activation, followed by cycles of 15 s at 95 °C, 15 s at the annealing temperature, and 60 s at 72 °C. Primers for IL-1β, IL-12, MCP-1, TNF-α, and glyceraldehyde-3-phosphate dehydrogenase (GAPDH) were obtained from Bioneer (Daejeon, Republic of Korea). Gene expression was quantified using the comparative Ct (ΔΔCt) method, with GAPDH as the endogenous control.

### 4.6. Statistical Analysis

Data are presented as mean ± standard error of the mean (SEM). Mice were allocated to groups as described in Section 4.3 (control and MIT-001 groups, *n* = 4 each; DSS and DSS + MIT-001 groups, *n* = 8 each); the number of animals analyzed for each outcome, after accounting for the deaths and occasional technical sample losses noted in Section 4.3, is reported in the corresponding figure legends. The histological injury score served as the principal in vivo outcome. Two-group comparisons were performed using the Mann–Whitney U test in view of the modest sample size and non-Gaussian distribution of several variables. Comparisons among three or more groups were performed using the Kruskal–Wallis test followed by Dunn’s post hoc test, or one-way analysis of variance (ANOVA) followed by Tukey’s post hoc test where assumptions of normality and equal variance were met. Two-sided *p* values less than 0.05 were considered statistically significant. No formal correction for multiple comparisons across cytokine endpoints was applied; therefore, cytokine results other than IL-1β should be interpreted as exploratory. All statistical analyses were performed using SPSS Statistics version 24.0 (IBM Corp., Armonk, NY, USA).

## 5. Conclusions

The present study demonstrates that MIT-001, a mitochondria-targeted ROS scavenger with anti-necrotic activity, reduces hydrogen peroxide-induced necrotic cell death in intestinal epithelial cells and ameliorates acute DSS-induced colitis in mice. These effects are associated with reduced HMGB1 expression and decreased IL-1β expression in inflamed colonic tissue. Although the present data do not establish a definitive causal HMGB1-mediated mechanism, they support the concept that pharmacological inhibition of mitochondrial ROS-driven regulated necrotic cell death may serve as an adjunctive upstream strategy in IBD. Further studies incorporating direct mechanistic interrogation (HMGB1 blockade and rescue; RIPK3/MLKL and ferroptosis markers; nuclear, cytoplasmic, and extracellular HMGB1 quantification), chronic and immune-dysregulation colitis models, dose–response evaluation, and pharmacokinetic and microbiota profiling will be required to delineate the therapeutic potential of MIT-001 in human IBD.

## Figures and Tables

**Table 1:** Histologic scoring criteria for colitis.

Feature	Grade	Description
Inflammation	0	None
	1	Slight
	2	Moderate
	3	Severe
Extent	0	None
	1	Mucosa
	2	Submucosa
	3	Transmural
Regeneration	0	Complete regeneration
	1	Almost complete regeneration
	2	Regeneration with crypt depletion
	3	Surface epithelium not intact
	4	No tissue repair
Crypt damage	0	None
	1	Basal 1/3 damaged
	2	Basal 2/3 damaged
	3	Only surface epithelium intact
	4	Entire crypt and epithelium lost
Percent involvement	1	1–25%
	2	26–50%
	3	51–75%
	4	76–100%

## Data Availability

The minimal dataset supporting the conclusions of this article is included as Supplementary Information (Dataset S1). Additional data generated and analyzed during the current study are available from the corresponding author upon reasonable request.

## Supplementary Materials

The following supporting information can be downloaded at: https://www.mdpi.com/article/10.3390/ijms27136051/s1.

## Abbreviations

The following abbreviations are used in this manuscript:

DAMP	damage-associated molecular pattern
DSS	dextran sulfate sodium
GPX4	glutathione peroxidase 4
GAPDH	glyceraldehyde-3-phosphate dehydrogenase
HMGB1	high-mobility group box 1
IBD	inflammatory bowel disease
IEC	intestinal epithelial cell
IL	interleukin
MCP-1	monocyte chemoattractant protein-1
MLKL	Mixed-lineage kinase domain-like protein
PARP-1	poly(ADP-ribose) polymerase-1
PCR	polymerase chain reaction
PI	propidium iodide
RIPK3	receptor-interacting protein kinase 3
ROS	reactive oxygen species
TLR	Toll-like receptor
TNF-α	tumor necrosis factor-α
