# Supplementary material for: MIT-001, a Mitochondria-Targeted ROS Scavenger, Ameliorates DSS-Induced Colitis and Is Associated with Reduced HMGB1 and IL-1β Expression
Source: Int J Mol Sci. 2026 Jul 6;27(13):6051. doi: 10.3390/ijms27136051 (PMC13360840; doi:10.3390/ijms27136051)
Supplement: Supplementary file 1 [file ijms-27-06051-s001.zip › Figure_S1.pdf]

Figure S1. Original, uncropped images.

Manuscript ijms-4373139. These are the original, unprocessed image files underlying the representative panels shown in the main figures.

(a) Western blot (corresponds to Figure 1B): PARP-1

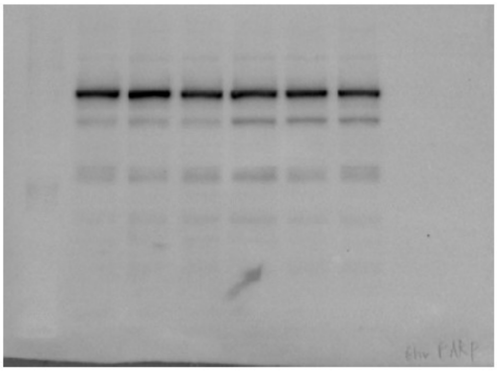

PARP-1 blot: full-length (116 kDa), 89-kDa apoptotic fragment, and 55-kDa necrotic fragment. Six lanes.

(b) Western blot (corresponds to Figure 1B): GAPDH loading control

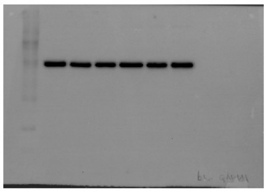

GAPDH loading control for the same samples.

(c) HMGB1 immunohistochemistry (corresponds to Figure 3A)

Representative HMGB1 IHC images of colonic tissue. Brown (DAB) indicates HMGB1-positive cells; blue (hematoxylin) indicates nuclei. All panels acquired at the same magnification.

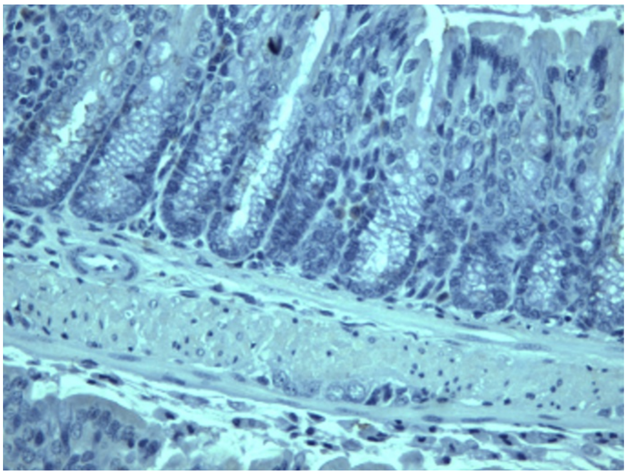

Control

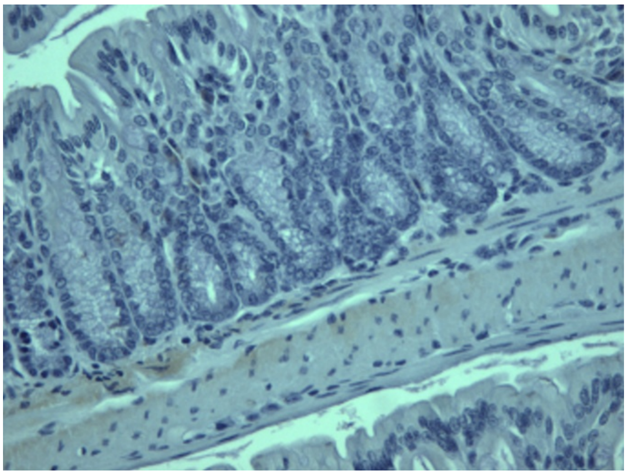

MIT-001

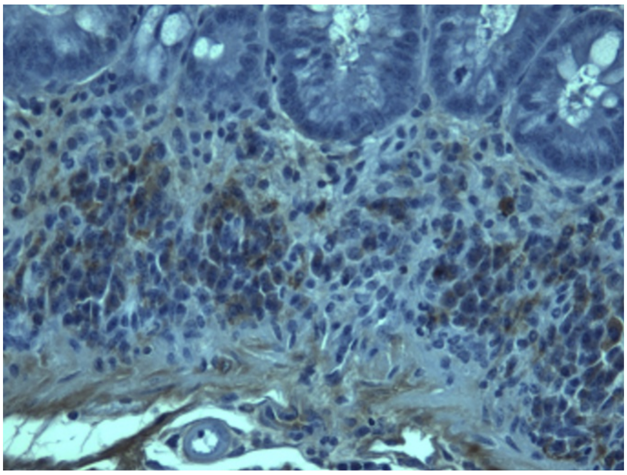

DSS

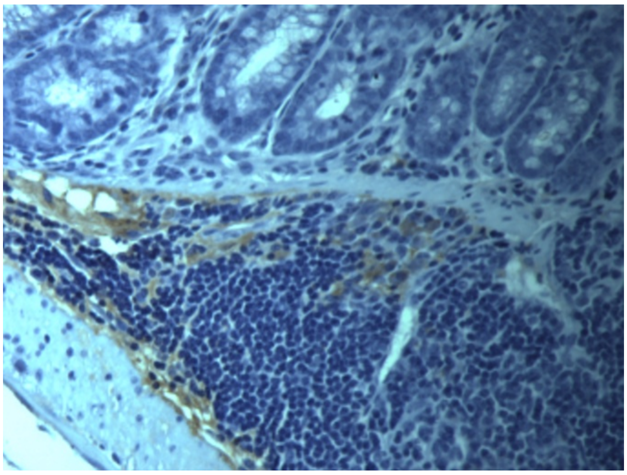

DSS + MIT-001
